# Selective *in vivo* and *in vitro* activities of 3,3′-4-nitrobenzylidene-bis-4-hydroxycoumarin against methicillin-resistant *Staphylococcus aureus* by inhibition of DNA polymerase III

**DOI:** 10.1038/srep13637

**Published:** 2015-09-01

**Authors:** Zheng Hou, Ying Zhou, Jing Li, Xinlei Zhang, Xin Shi, Xiaoyan Xue, Zhi Li, Bo Ma, Yukun Wang, Mingkai Li, Xiaoxing Luo

**Affiliations:** 1 Department of Pharmacology, School of Pharmacy, Fourth Military Medical University, Xi’an, Shaanxi, 710032, China; 2 School of Chemical Engineering, Xi’an University, Xi’an, Shaanxi, 710065, China; 3 School of Pharmacy, Fourth Military Medical University, Xi’an, Shaanxi, 710032, China

## Abstract

As the persistent resistance of *Staphylococcus aureus* to available antibiotics is associated with high infection incidence, mortality rate and treatment cost, novel antibacterial agents with innovative therapeutic targets must be developed. 3,3′-(4-Nitrobenzylidene)-bis-(4-hydroxycoumarin) (NBH), a dicoumarin derivative, was reported to exert antibacterial activity. This study investigated the underlying mechanisms of *in vivo* and *in vitro* activities of NBH against *S. aureus*. NBH exerted bactericidal effects against the tested *S. aureus* and *Staphylococcus epidermidis* strains *in vitro*, with low cytotoxicity and resistance and high plasma stability. NBH also exhibited therapeutic effects *in vivo* on septicaemic mice. Results of molecular docking and analysis on morphological change, DNA production and polymerase inhibition suggested that DNA polymerase could be the target of NBH. These findings indicated that dicoumarin derivatives, which interfere with DNA replication, could be developed as a potential agent against *S. aureus*, particularly methicillin-resistant strains.

Methicillin-resistant *Staphylococcus aureus* (MRSA), one of the most common causes of bloodstream infection^1^,^2^, is associated with severe invasive infections^3^ and high mortality rates^4^. Currently, vancomycin remains the “last-resort” antibiotic against MRSA but its use in the United States and Europe has been associated with therapeutic failures caused by persistent MRSA^5^,^6^. Although development of linezolid has contributed to MRSA treatment, a clinical outbreak of linezolid-resistant *S. aureus* was reported in an intensive care unit^7^. The rapid development of bacterial resistance to multiple antibiotics has compelled the need to immediately improve currently used antibacterial strategies and develop new antibiotics with different chemical structures from traditional antibiotics.

Coumarin derivatives are plant-derived or chemically synthesised products known for their broad biological activities^8^. Li *et al.*^9^ reported that some coumarin derivatives could inhibit the replicative DNA helicase of *S. aureus*. Dicoumarol, a coumarin derivative biosynthesised from *o*-coumaric acid and 4-hydroxycoumarin, exhibited strong anticoagulant activity *in vivo*. Previous studies confirmed that 3,3′-(4-nitrobenzylidene)-bis-(4-hydroxycoumarin) (NBH), a dicoumarol derivative, exerted antibacterial activities against *S. aureus in vitro*^10^. Nevertheless, few works have demonstrated the *in vivo* activity of NBH in animal models. The antibacterial mechanism of NBH against MRSA also remains unclear.

This study investigated the antibacterial action of NBH *in vivo* and the associated mechanism against *S. aureus*. NBH and five other dicoumarin derivatives were synthesised, and their antibacterial activity was observed in methicillin-susceptible *S. aureus* and MRSA strains *in vitro*. The reliability and efficacy of NBH treatment were also evaluated in BALB/c mice infected with MRSA *in vivo*. Morphological methods, DNA polymerase assay and molecular docking study were used to explore the mechanism of action of NBH.

## Results

### Drug-likeness evaluation

The results showed that all six compounds (BH, NBH, TBH, MBH, MOBH and DBH) obeyed Lipinski’s rule of five (Table 1).

### Bacterial susceptibility testing and growth assay

NBH exerted antibactericidal effect against the four tested *S. aureus* (Table 1) and two *Staphylococcus epidermidis* strains (Supplementary Table 1), including methicillin-susceptible and -resistant strains. NBH presented minimum inhibitory concentration (MIC) values that ranged from 16 mg/L to 32 mg/L. NBH exerted no effects against Gram-negative strains, with MIC values of more than 256 mg/L for *Escherichia coli* ATCC25922 and *Pseudomonas aeruginosa* ATCC27853 (Supplementary Table 1).

**Figure 1:**
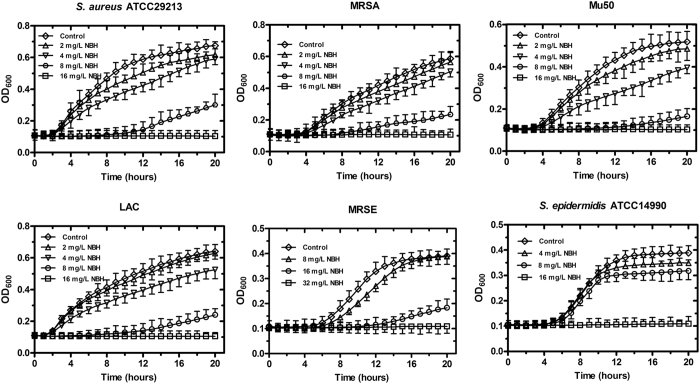
Concentration-dependent inhibition of NBH on the growth of four *S. aureus* and two *S. epidermidis* strains. NBH was added to cell cultures containing different tested strains to final concentrations of 2, 4, 8, 16 or 32 mg/L, with addition of an equal volume of sterile water as the controls. The growth curves for six tested strains were measured using a BioscreenC™ instrument in the absence and presence of different concentrations of NBH. Sample frequency was 1 h, and data at specified time points are represented as mean ± SD for three replicates.

To evaluate the growth inhibitory effects of NBH on four *S. aureus* and two *S. epidermidis* strains, we added different concentrations of the compound to the cultures. NBH concentration-dependently inhibited the growth of the pathogens and almost completely inhibited the growth of *S. aureus* and *S. epidermidis* ATCC14990 at 16 mg/L and methicillin-resistant *S. epidermidis* (MRSE) at 32 mg/L (Fig. 1).

**Figure 2:**
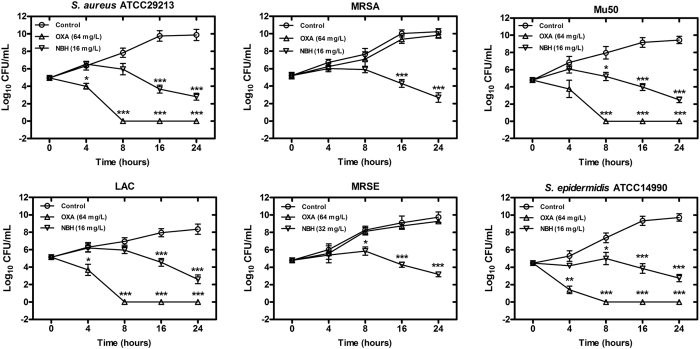
Effects of NBH on the growth of bacterial colonies. NBH and oxacillin were added to cell cultures to final concentrations of 16 (32 mg/L to MRSE) and 64 mg/L, with addition of equal volumes of sterile water as the controls. Aliquots of each culture were collected at 0, 4, 8, 16 and 24 h, diluted and inoculated on solid agar. CFUs were calculated from the number of colonies grown on plates, and data are represented as mean ± SD for three replicates. *P < 0.05, **P < 0.01 and ***P < 0.001 versus control; OXA, oxacillin.

The antibacterial effects of NBH were evaluated through cell viability assays. NBH exerted time-dependent bactericidal effects on all tested pathogens. Treatment with 16 and 32 mg/L NBH decreased the colony-forming units (CFUs) of *S. aureus* and *S. epidermidis* from the initial values of 10^5^ CFU/mL (Fig. 2). After treatment for 24 h, the bactericidal effects reduced and 10^2^ CFU/mL viable bacteria were observed. By contrast, 64 mg/L oxacillin reduced the CFUs of *S. aureus* ATCC29213, Mu50, USA 300 (Los Angeles County clone, LAC) and *S. epidermidis* ATCC14990 from 10^5^ CFUs/mL to <10 CFUs/mL within 8 h (Fig. 2). Nevertheless, oxacillin did not inhibit the growth of MRSA and MRSE at the concentration of 64 mg/L.

### Cytotoxicity *in vitro*

**Figure 3:**
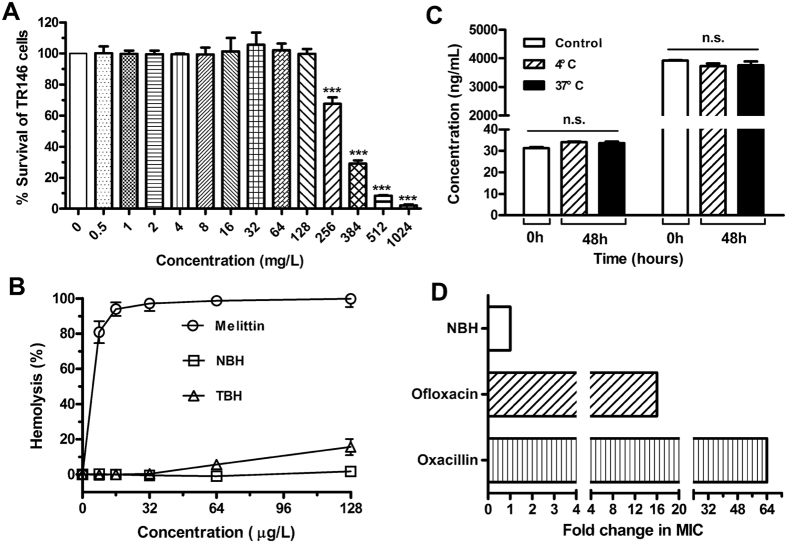
*In vitro* properties of NBH. (**A**) Cell toxicity of 0.5 mg/L to 1024 mg/L NBH determined on TR146 cells after 24 h of incubation. Each plot was obtained from a representative experiment, and data points are represented as mean ± SD of three replicates. ***P < 0.001 versus control. (**B**) Haemolytic toxicities of NBH (squares), TBH (triangles) and melittin (circles) determined in human red blood cells (2% haematocrit) after 1 h of incubation. Data are shown as mean ± SD values for six samples. (**C**) Quantification of NBH after preincubation with 95% rat plasma at 4 °C or 37 °C for 48 h. Data are represented by mean ± SD of six replicates. n.s. = *P* > 0.05 versus control. D. Emergence of resistance in *S. aureus* ATCC29213 after 15 serial passages in the presence of antimicrobials. Relative MIC is the normalised ratio of MIC obtained for the 15th subculture to MIC obtained upon first exposure.

To explore the selectivity and safety of NBH, we investigated its cytotoxicity to TR146 cells and human red blood cells (hRBCs) *in vitro* by using the 3-(4,5-dimethyl-2-thiazyl)-2,5-diphenyl-2H-tetrazolium bromide (MTT) method and haemolytic assay, respectively. MTT data showed that NBH exhibited cytotoxicity against TR146 cells at concentrations higher than 256 mg/L (Fig. 3A), which is ten times higher than the MIC for *S. aureus*. NBH did not affect hRBC viability at 8 mg/L to 128 mg/L compared with melittin (GIGAVLKVLTTGLPALISWIKRKRQQ), an antimicrobial peptide that exhibits powerful lytic activity against bacterial and eukaryotic cells (Fig. 3B). However, 128 mg/L TBH demonstrated significant cytotoxicity in hRBCs (Fig. 3B).

### Plasma stability

NBH concentration did not significantly change after preincubation with rat plasma at 4 °C and 37 °C for 48 h (Fig. 3C).

### Induction of resistance

After 24 h of incubation at 37 °C, NBH-treated groups presented no resistant colonies. Therefore, the calculated frequency of resistance to NBH was lower than 10^−9^. After 15 serial passages of *S. aureus* ATCC 29213 in the presence of sub-MIC, the relative MIC values of NBH remained constant, which indicated that antibiotic resistance was not induced (Fig. 3D). However, the relative MIC values of ofloxacin and oxacillin increased by 16- and 64-fold, respectively.

### Antibacterial activity *in vivo*

**Figure 4:**
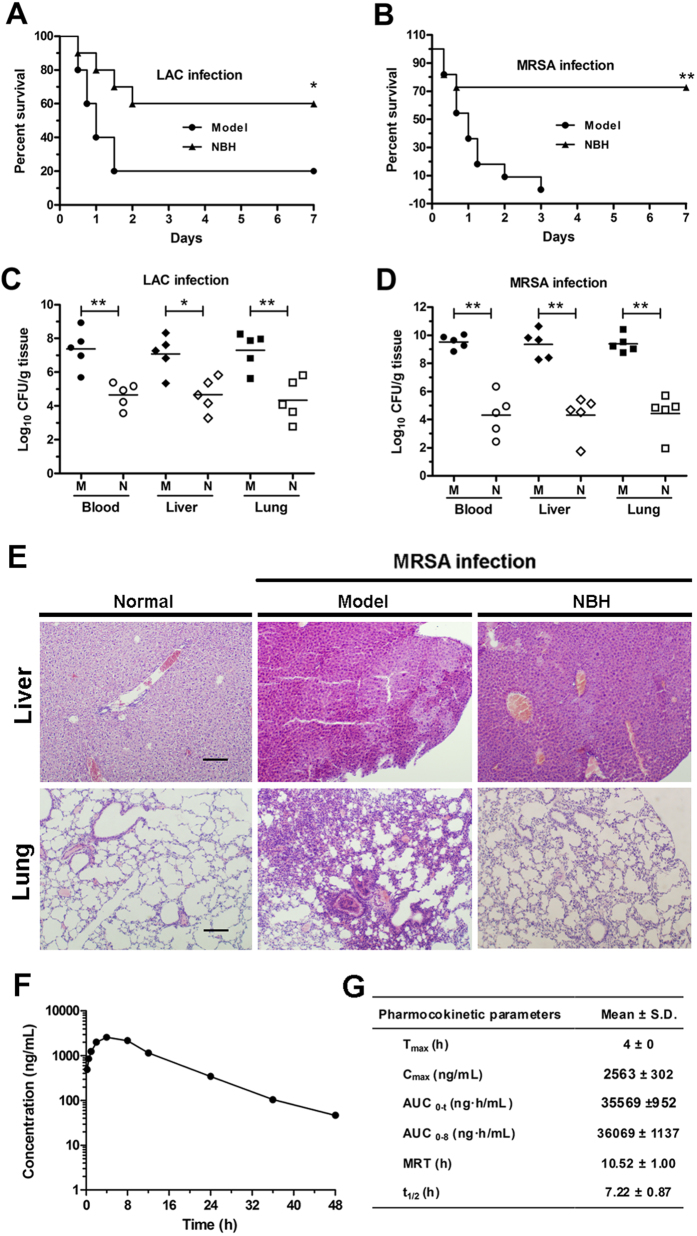
*In vivo* activities of NBH. (**A**) Survival of BALB/c mice (*n* = 10/group) inoculated by i.p. injection with LAC and treated with 5 mg/kg NBH containing 0.5% sodium carboxymethylcellulose by intragastric administration at 1 and 6 h after infection. (**B**) Survival of BALB/c mice (*n* = 11/group) inoculated by i.p. injection with MRSA and treated with 5 mg/kg NBH containing 0.9% saline by i.p. administration at 1 and 6 h after infection. (**C**) Colonisation of LAC inoculum in the liver, lung and blood cultures of NBH-treated BALB/c mice (n = 5/group) 24 h after infection. (**D**) Colonisation of MRSA inoculum in the liver, lung and blood cultures of NBH-treated BALB/c mice (n = 5/group). M, model group; N, NBH-treated group. *P < 0.05, **P < 0.01 versus model. (**E**) Morphologies of livers and lungs were examined with H&E staining in BALB/c mice (original magnification, 100×). Normal, uninfected BALB/c mice; Model, BALB/c mice infected with MRSA; NBH, BALB/c mice infected with MRSA and treated with 5 mg/kg NBH. Scale bars represent 200 μm. (**F**) Quantification of NBH concentrations in rat blood after intragastric administration at 7 mg/kg dosage to rats (n = 3). Mean plasma concentrations of NBH in rats at 0.25, 0.5, 1, 2, 4, 8, 12, 24, 36 or 48 h after administration. G. Pharmacokinetic parameters of NBH.

To test whether NBH could provide a survival benefit *in vivo*, we performed challenge experiments with LAC or MRSA. Results showed that NBH significantly improved the animal survival in the LAC and MRSA groups compared with that in the model group (Fig. 4A,B and Supplementary Fig. 1A). As survival is related to reductions in bacterial titres in mouse tissues (liver, lung and blood), we measured CFUs in the treatment groups. After NBH treatment, mice that survived lethal LAC or MRSA infection demonstrated an average of three to four log units decrease (in CFU) in liver, lung and blood (Fig. 4C,D and Supplementary Fig. 1B).

Pathological change was also observed in MRSA-infected mice after NBH treatment. The results showed that the normal alveolar structure disappeared in MRSA-infected mice in the model group (Fig. 4E). Congestion, necrosis or infiltration and accumulation of a large number of neutrophils appeared in lung tissues. Some neutrophils infiltrated the local alveolar space and alveolar septum, and partial alveolar fusion was observed in NBH-treated group. Swelling of liver cells and haepatic sinusoidal dilation and congestion were observed in the model group (Fig. 4E), whereas these pathological changes were less pronounced in NBH-treated group.

### Pharmacokinetic study

The pharmacokinetic properties of NBH was evaluated in male SD rats. Analysis of blood after single-dose intragastric administration revealed that the plasma concentration of NBH rapidly increased, with a half-life of 7.2 h. The pharmacokinetic profile showed that NBH was stable, and persisted in the bloodstream for 48 h (Fig. 4F). The pharmacokinetic parameters evaluated are shown in Fig. 4G.

### Morphological change *in vitro*

**Figure 5:**
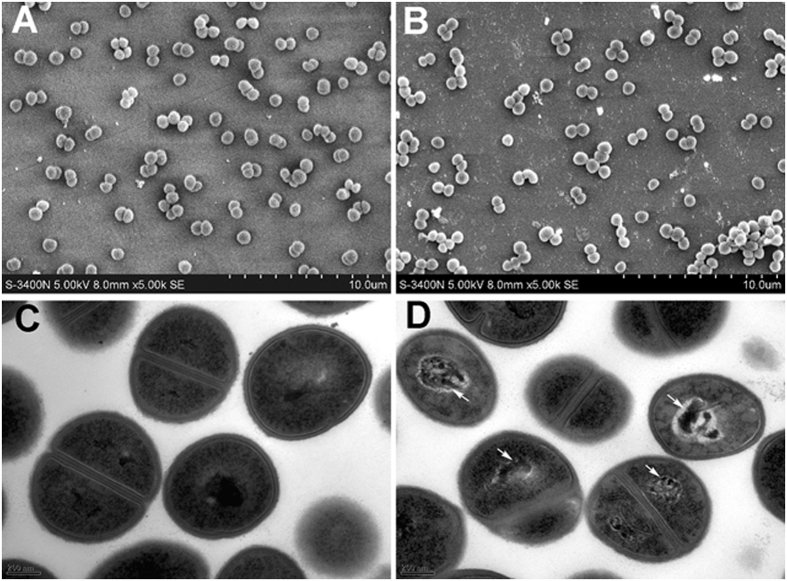
Morphologies of LAC investigated through SEM and TEM at 90 min after treatment with 24 mg/L NBH. (**A**) Control (SEM); (**B**) LAC treated with 24 mg/L NBH for 90 min (SEM). (**C**) Control (TEM); D. LAC treated with 24 mg/L NBH for 90 min (TEM). White arrows indicate bacterial cells with condensed DNA molecules.

To further explore the possible antibacterial mechanism of NBH, we observed the morphological change of MRSA through scanning electron microscopy (SEM) and transmission electron microscopy (TEM). After treatment with 24 mg/L NBH for 90 min, the SEM images revealed no remarkable change in bacterial morphology (Fig. 5A,B). However, the TEM images showed that an electron-light region appeared in the centre of the cells and separated on the nucleoid zone from the surrounding cytoplasm after NBH treatment for 90 min; this region contained condensed DNA molecules (arrow in Fig. 5D). The cytoplasm became amorphous and lost the ‘‘grainy’’ appearance typical for intact cells (Fig. 5D). By contrast, a comparatively apparent nuclear region appeared in the centre of normal *S. aureus* cells (Fig. 5C).

### Inhibition of NBH on genomic DNA production^11^,^12^

**Figure 6:**
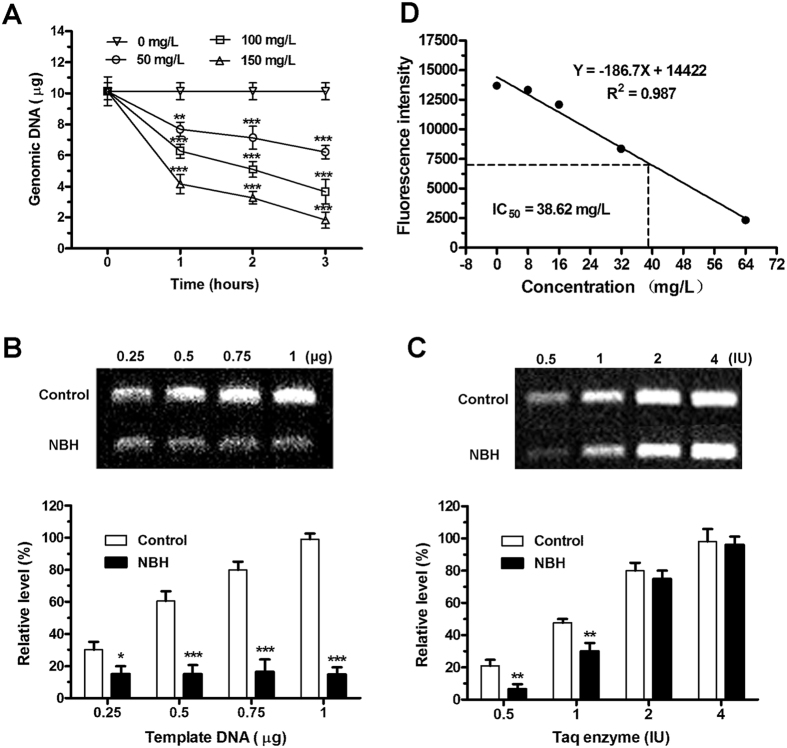
Antibacterial mechanism of NBH. (**A**) Inhibition of NBH on genomic DNA production. NBH was added to cell cultures containing LAC to a final concentration of 50, 100 or 150 mg/L. Aliquots of each culture at 240 rpm were collected at 0, 1, 2, 3 and 4 h and then harvested and lysed. Genomic DNA was extracted and quantified from the bacterial lysis mixture. Data are shown as mean ± SD (*n* = 3). **P < 0.01, ***P < 0.001 versus control. B. Increase in template DNA could not reverse polymerisation inhibition by NBH. Genomic DNA was extracted for LAC and used as the template. PCR was performed using NBH (32 mg/L), DNA (0.25, 0.5, 0.75 or 1 μg) and Taq enzyme (0.5 IU) for 16 cycles. The amplified products were visualised on 1% agarose gels and quantified. Data are shown as mean ± SD (*n* = 3). *P < 0.05, ***P < 0.001 versus control. C. Increase in Taq enzyme could reverse polymerisation inhibition by NBH. Genomic DNA was extracted for LAC and used as the template. PCR was performed using NBH (32 mg/L), DNA (0.25 μg) and Taq enzymes (0.5, 1, 2 or 4 IU) for 16 cycles. The amplified products were visualised on 1% agarose gels and quantified. Data are shown as mean ± SD (*n* = 3). **P < 0.01 versus control. D. DNA polymerase inhibition by NBH. NBH was added to the reaction mixture containing fluorescence dye to final concentrations of 0, 8, 16, 32 or 64 mg/L. Fluorescence intensity of DNA production was determined. Data are shown as mean ± SD (*n* = 3). *R*^2^ represents the correlation coefficient of assay linearity. IC_50_ was calculated based on the linear equation.

To determine the effects of NBH on the genomic DNA of LAC, we applied 50, 100 and 150 mg/L NBH to *S. aureus* cultures. Genomic DNA was isolated, and absorbance was determined at 260 nm. Figure 6A shows that NBH time-dependently reduced the production of genomic DNA. Treatment with 50, 100 and 150 mg/L NBH significantly affected genomic DNA production. These results indicated that NBH could effectively influence the quantity of genomic DNA.

### Polymerisation inhibition

Polymerisation strength is mainly dependent on the amount of DNA template and polymerase. To understand the mechanism of NBH on polymerisation inhibition, we added different amounts of template or polymerase for polymerase chain reaction (PCR). The results showed that amplification of 16S rRNA improved with increasing DNA template in the control group, but no changes was observed in the NBH treatment group (Fig. 6B). Four amounts of Taq enzymes (0.5, 1, 2, and 4 U) could effectively reverse NBH inhibition on amplification of 16S rRNA compared with that in the control group (Fig. 6C). The relative amplification levels of 16S rRNA in NBH-treated *S. aureus* were 31.76%, 63.03%, 93.75% and 97.66%, with increasing amount of Taq enzyme. These data indicated that NBH may interfere with the activity of DNA polymerase.

### DNA polymerase inhibition

To ascertain whether NBH could repress the activity of DNA polymerase, we assayed activation of the enzyme. Our data demonstrated that the fluorescence intensity of DNA production decreased with increasing NBH concentration (Fig. 6D). Moreover, a linear correlation existed between NBH concentration and DNA polymerase inhibition (*R*^2^ for linear fit = 0.987). These results showed that NBH could repress the activity of DNA polymerase and function as DNA polymerase inhibitor (IC_50_ = 38.62 mg/L).

### Molecular docking

**Figure 7:**
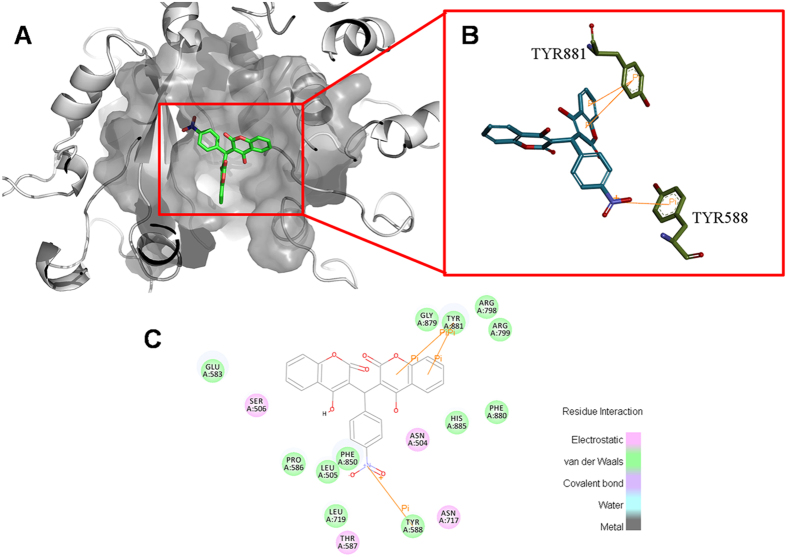
Binding site interaction was predicted through docking study in PolC. (**A**) Spatial binding analysis of NBH with PolC. (**B**) 3D interaction of NBH with TYR588 and TYR881 of PolC. (**C**) 2D relation diagram of NBH and all amino acid residues in PolC.

Docking results showed that NBH shared a very similar binding pocket to dGTP in the complex. The 3D schematic (Fig. 7A,B) showed three noticeable pi–pi interactions between the benzene rings of NBH and two amino acids (TYR881 and TYR558) of polC in the active site. This binding conformation can be stabilised by the interaction between NBH hydrophobic backbone and four hydrophobic amino acid residues (PHE880, HIS885, LEU505 and LEU719) around the active pocket (Fig. 7C). These binding characteristics are similar to the previously reported characteristics^13^. Hydrogen bonds between the docked pose and polC were not observed (Fig. 7).

## Discussion

As *S. aureus* remains a leading cause of nosocomial infection worldwide^14^,^15^, traditional therapy may be insufficient in most patients and antibiotics with novel structure or target must be developed to fight MRSA. Our data showed that five dicoumarin derivatives (BH, NBH, TBH, MBH and MOBH) exerted different anti-*S. aureus* activities. The activities of TBH and NBH were more effective than that of the other compounds. However, cytotoxicity results indicated that NBH was safer than TBH. The calculated CFUs showed that the bactericidal rate of NBH was lower than that of oxacillin.

Sepsis is a serious and potentially lethal complication of *Staphylococcus* infection^4^. The present results showed that NBH exhibited more effective therapeutic effects against *S. aureus* i.p. challenge by i.p. injection than through oral administration. NBH significantly decreased the bacterial counts in both modes of administration. Furthermore, histological experiments showed that NBH could effectively suppress the bacterial titres in the blood, liver and lung tissues of MRSA-infected mice compared with those in control-infected mice. Hence, NBH could alleviate pathological changes associated with extensive lung and liver damages.

Although vancomycin is the most frequently used antibiotic for MRSA, its reduced susceptibility to MRSA and toxicity have restricted its clinical applications^16^,^17^, thereby considerably hampering its clinical development and systemic applications. This study showed that NBH exhibited minimal toxicity on eukaryotic cells. Furthermore, resistance frequency test and multigenerational induced resistance experiment were conducted. The results showed that the propensity of NBH for innate and induced resistance was very low. In addition, plasma stability and pharmacokinetic studies revealed that NBH demonstrated good plasma stability *in vitro* and *in vivo*. These findings, as well as the protective antibacterial activity exerted by NBH *in vivo*, provide useful information for development of NBH as a novel compound for treatment against *Staphylococcus* infection. Nevertheless, the structure and antibacterial activity of NBH must be optimised. Hence, in-depth studies are needed to explore the mechanism of action and specific target of NBH for development of promising compounds with similar structures.

After NBH treatment, an electron-light region, which contained numerous condensed DNA molecules, was observed through TEM in the centre of the LAC cytoplasm. As DNA replication is effectively conducted under the relaxed state, we speculated that NBH exerted antibacterial effect by interfering DNA replication and decreasing the amount of DNA. To prove our hypothesis, we determined the amount of genomic DNA of LAC cultured with different NBH concentrations for 1, 2 and 3 h. NBH time- and concentration-dependently decreased the genomic DNA amount of LAC.

We further speculated that DNA replication was inhibited, thereby decreasing the amount of DNA in NBH-treated LAC. As polymerase and template are the two key factors in DNA replication, we confirmed the effect of NBH in DNA replication by performing PCR to simulate the replication process of bacteria. Different quantities of DNA and Taq enzymes were added to the PCR mixture. The results showed that polymerisation inhibition by NBH was not reversed by the increase in DNA template but considerably reversed by the increase in Taq enzyme. These data indicated that NBH may exert antibacterial effect by inhibiting the activity of polymerase.

To confirm that NBH repressed the activity of DNA polymerase, we performed enzymatic assay using ProFoldin DNA polymerase III. The results showed that the activity of DNA polymerase III gradually declined with increasing NBH concentration. NBH concentration was linearly correlated with inhibition of DNA polymerase, with a correlation coefficient of 0.987. Molecular docking data also suggested that NBH may affect the function of DNA polymerase by competing with the substrate binding site of polC with dGTP. These data showed that NBH could specifically inhibit the activity of DNA polymerase III, and NBH could be a specific inhibitor of DNA polymerase III. As DNA is the most important genetic basis stored in cells, DNA structural abnormalities, in a condensed form, inevitably lead to mutation or death of bacteria. The condensed form of DNA may be due to the manifestation on its impaired replication. Inhibition of DNA polymerase and severe interference of DNA replication by NBH may sharply decline the DNA quantity, resulting in formation of an electron-light region in LAC.

Only few new classes of antibiotics, including lipopeptides^18^, oxazolidinones^19^ and streptogramins^20^, with targets that differed from those of traditional antibiotics, have been clinically applied over the past 50 years^21^. Hence, novel therapeutic targets for treatment of drug-resistant bacteria infections must be investigated. DNA polymerase varies considerably between eukaryotes (α, β, γ, δ and ε subtypes) and prokaryotes (I, II, III, IV and V subtypes) and exhibits high conservation in bacteria; hence, this enzyme could be an ideal therapeutic target for bacterial infections^22^. Several polymerase inhibitors have been found^23^,^24^, but antibiotics targeting bacterial DNA polymerase have not been developed. Our data suggests that replicative DNA polymerase may be the target of NBH and its derivatives. The structure of NBH and its derivatives must be further modified to develop potent and promising agents for treatment of infections caused by *Staphylococcus*, particularly multi-drug resistant *Staphylococcal* strains.

## Methods

### Drug-likeness evaluation

The Lipinski rule of five^13^,^25^ defines four simple physicochemical parameter ranges (molecular weight [MWt] ≤ 500, log *P* ≤ 5, H-bond donors ≤ 5 and H-bond acceptors ≤ 10). The six compounds were predicted via Lipinski drug filter.

### Strains and chemicals

*S. aureus* ATCC29213, *S. epidermidis* ATCC14990, *E. coli* ATCC25922 and *P. aeruginosa* ATCC27853 were purchased from the Chinese National Centre for Surveillance of Antimicrobial Resistance. MRSA XJ75302 and MRSE XJ75284 were isolated from the clinical laboratory of Xijing Hospital (Xi’an, China). Mu50 (ATCC700699) was purchased from MicroBiologics (Saint Cloud, MN, USA). LAC (USA300) strain was provided by Michael Otto and identified through 16S rRNA gene sequencing by Qingdao Qibiao Testing Co., Ltd.

Oxacillin, ofloxacin and levofloxacin were purchased from the National Institute for the Control of Pharmaceutical and Biological Products (Beijing, China). Six dicoumarin derivatives (Table 1) were synthesised according to previously reported methods^10^.

On the basis of the standards of the Clinical and Laboratory Standards Institute (CLSI) for dissolution of ceftobiprole^26^, we completely dissolved 3.0 mg of NBH in 120 μL of dimethyl sulfoxide (DMSO). The solution was added with 20 μL of glacial acetic acid and vortexed vigorously. The solution was diluted to 1.024 mg/mL by using distilled water.

### Bacterial susceptibility testing

MIC values were determined through microdilution assay in sterilised 96-well polypropylene microtiter plates according to the broth microdilution guidelines of CLSI^27^. Triplicate bacterial cells in logarithmic growth phase were diluted to 5 × 10^5^ CFU/mL and then incubated with NBH, which was added to each tested strain culture to final concentrations of 0, 2, 4, 8, 16 or 32 mg/L. The strains were cultivated in an automated Bioscreen C system (Labsystems Helsinki, Finland) by using Mueller–Hinton (MH) broth. Time-kill curves for *S. aureus* and *S. epidermidis* were determined using drop plate method according to the basic microbiological protocols^28^.

### Cytotoxicity assay

Cytotoxicity of NBH to TR146 cells, which originated from a human neck metastasis of buccal carcinoma, was determined through standard MTT assay according to a previously established method^29^,^30^. TR146 cells were seeded in 96-well microtitre plate at a density of 1 × 10^4^ cells/well. After incubating the cells with various concentrations of NBH for 24 h, MTT was performed and the percentage of cell survive was then determined.

Haemolysis was evaluated by determining the amount of haemoglobin released in 2% suspensions of fresh human erythrocytes^31^. Subsequently, 100 μL aliquots of hRBC suspensions were added to a 96-well microtiter plate containing NBH, TBH or melittin at 8, 16, 32, 64 and 128 mg/L for 1 h at 37 °C. The samples were centrifuged, and haemolytic toxicity was assessed as a function of haemoglobin leakage by determining the absorbance of 200 μL of supernatant at 540 nm.

### Plasma stability

Approximately 30 or 4000 ng/mL NBH was preincubated with rat plasma at 4 °C or 37 °C for 48 h. After incubation, the samples were analysed through LC/MS/MS. Statistical data were obtained from six independent experiments performed.

### Resistance studies

For single-step resistance, *S. aureus* ATCC29213 at 10^9^ CFU was plated on MHA containing 2×, 4×, 8× and 16× MIC of NBH^32^,^33^.

For resistance development by sequential passaging^31^,^33^, *S. aureus* ATCC29213 cells at the exponential phase were diluted to an A_600_ nm of 0.01 in 1 mL of MHB containing NBH or ofloxacin. The cells were incubated at 37 °C with agitation and passaged at 24 h intervals in the presence of NBH, oxacillin or ofloxacin at sub-inhibitory concentration. MIC was determined through broth microdilution.

### Ethics statement

All mouse experiments and animal care procedures were reviewed and approved by the Animal Resource Centre of the Fourth Military Medical University. And the methods were carried out in strict accordance with the approved guidelines.

### Bacterial challenge and NBH therapy

Specific-pathogen-free male BALB/c mice with body weights between 18 and 20 g were used in this study. The mice were infected by i.p. administration of 4.2 × 10^8^ CFU LAC or 7.8 × 10^9^ CFU MRSA XJ75302 inoculum in 0.4 mL of MH broth. In the LAC-infected model, the mice (n = 15/group) were randomised to receive 5 mg/kg of NBH (20 ml/kg, containing 0.5% sodium carboxymethylcellulose) via intragastric administration after bacterial challenge for 1 h and 6 h. In the MRSA-infected model, mice (n = 16/group) received i.p. injection 5 mg/kg of NBH (20 ml/kg, containing 0.9% saline) at 1 h and 6 h post infection. To assess bacterial clearance, 5 mice in each group were removed from their cages and 100 μL blood samples for culture were obtained from the tail vein through aseptic percutaneous puncture 24 h after infection. Then cervical dislocation was performed manually resulting in euthanasia within 10 seconds, and liver and lung were harvested aseptically from these mice, weighed, and homogenized in sterile saline solution. Colony counts in these tissues were expressed as CFU/g of the tissue. The survival of 10 or 11 mice in each group was monitored for 7 d after infection, and the cumulative percentage survival was determined. In the MRSA infection model, parts of liver and lung were fixed in 10% neutral buffered formalin for 24 h. Morphology was observed using haematoxylin and eosin (H&E) staining.

### Pharmacokinetic analysis

A single dose of 7 mg/kg NBH (7 ml/kg, containing 0.5% sodium carboxymethylcellulose) was intragastrically administered to three normal pathogen-free SD rats. Blood samples (0.3 mL) were collected at specified time points via an indwelling catheter. The samples were then centrifuged for 10 min at 3000 × *g*. Subsequently, 100 μL of plasma aliquot was added to 300 μL of IS-precipitation mixture in a tube, vortexed for 30 s and centrifuged for 5 min at 14000 × *g*. Protein-free supernatant was analysed through LC/MS/MS using an API5000 triple quadrupole mass spectrometer (Applied Biosystems Inc., USA) coupled with a Shimadzu 20AD-XR UFLC system (Shimadzu, JAN).

### SEM and TEM

LAC (1.0 × 10^8^ CFU/mL) was cultured in 24 mg/L NBH at 120 rpm for 90 min, harvested and then washed. The specimens were observed through SEM (Hitachi S-3400N) or TEM (JEM-1230, JOEL), and images were recorded.

### Genomic DNA determination

LAC (5.0 × 10^8^ CFU/mL) was cultured with 0, 50, 100 or 150 mg/L NBH at 240 rpm for 1, 2 and 3 h, harvested and then washed. These cultures were centrifuged at 8000 × *g* for 10 min at 4 °C, and the supernatant was decanted. Cell pellet was suspended for 30 min at room temperature in 100 μL of lysis buffer with 1.5 mg of lysozyme and 0.1 mg of lysostaphin (Sigma–Aldrich, St. Louis, MO, USA). Genomic DNA was extracted from the bacterial lysis mixture by using TIANamp bacterial DNA kit (TIANGEN, Beijing, China) following the manufacturer’s instructions. DNA was quantified by determining absorbance with a spectrophotometer (DU800, Beckman, USA) at 260 nm.

### Polymerisation assay^11^,^12^

To detect suppression of NBH on DNA polymerisation, we performed PCR with 32 mg/L NBH, Taq Enzymes and DNA. The reaction conditions comprised 16 cycles for 30 s at 94 °C for denaturation; 30 s at 53 °C for annealing; and 45 s at 72 °C for extension. The forward (5′-CTTTATGGGATTTGCTTGA-3′) and reverse (5′-GTCGTGAGATGTTGGGTTA-3′) primers for 16S rRNA yielded a 216 bp product. Amplification ended with 5 min of incubation at 72 °C. The amplified products were visualised on 1% agarose gels.

### DNA polymerase assay

DNA polymerase assay was performed according to the instructions of ProFoldin DNA polymerase assay kit Plus (DPA100KN). The reaction mixture in a final volume of 40 μL consisted of 24 μL of NBH at different concentrations, 4 μL of 10 × buffer, 4 μL of 10 × DNA, 4 μL of 10 × *Streptococcus pneumoniae* DNA polymerase and 4 μL of 10 × dNTP mixtures. After the reaction mixture was incubated at room temperature for 60 min, 40 μL of the reaction mixture was added with 40 μL of 1 × fluorescence dye. Fluorescence intensity was determined at 535 nm by using the excitation wavelength at 485 nm after the mixture was incubated for 5 min.

### Homology modelling

The PolC/DPO3A (ID: P10443) sequence was retrieved from UniProt KB. the top-ranked template sequence, namely, *Geobacillus kaustophilus* PolC (Gka PolC, PDB ID:3F2B^34^), was selected by PSI-BLAST. More than 500 homology models were generated and optimised using the MODELLER module in Discovery Studio 3.5. The final 3D homology models of DPO3A were built according to their 3D structure and discrete optimised protein energy (DOPE) score.

### Molecular docking^35^,^36^

The 2D structures of small-molecule ligands were created using the ChemDraw panel. The 3D conformation of the ligands were generated and optimised with the Discovery Studio with the Merck molecular force field. The optimised energy and minimised conformation of DOPE were selected as the receptor for subsequent docking progress. The Charmm force field and all hydrogen atoms were added. The appropriate receptor model was identified using the minimisation module for 5000-step energy optimisation and Ramachandran plot analysis. In Discovery Studio, protein portion of the complex is defined as a receptor and dGTP as a ligand. Ligand-binding region was defined after comparison with the dGTP position and previously reported ligand-binding region^37^. After dGTP molecules was removed from the complex, docking was conducted by using the LibDock module and the results were analysed using Discovery Studio and PyMOL^38^.

### Statistical analysis

Results are expressed as mean ± SD. One-way and two-way ANOVA and Kaplan–Meier survival analysis were used for statistical evaluations. Statistical significance was defined at a probability value of *P *< 0.05.

## Additional Information

**How to cite this article**: Hou, Z. *et al.* Selective *in vivo* and *in vitro* activities of 3,3′-4-nitrobenzylidene-bis-4-hydroxycoumarin against methicillin-resistant *Staphylococcus aureus *by inhibition of DNA polymerase III. *Sci. Rep.* **5**, 13637; doi: 10.1038/srep13637 (2015).

## Supplementary Material

Supplementary Table S1 X Figure S1

## Figures and Tables

**Table 1:**
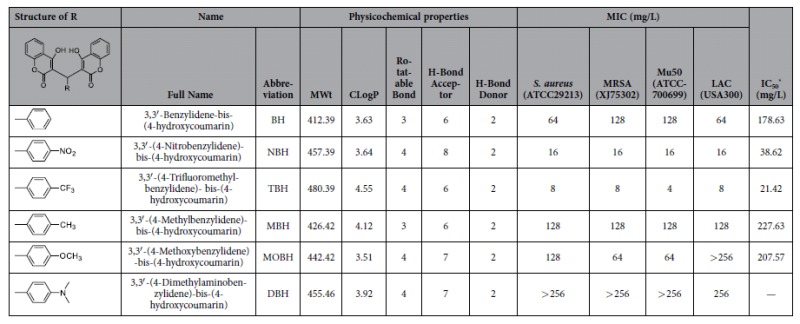
Structures, designations, physicochemical properties, activity on *S. aureus* and DNA polymerase inhibition of the compounds investigated.

^*^The compound concentration causing 50% inhibition of DNA polymerase activity.
