# Supplementary material for: Selective in vivo and in vitro activities of 3,3′-4-nitrobenzylidene-bis-4-hydroxycoumarin against methicillin-resistant Staphylococcus aureus by inhibition of DNA polymerase III
Source: Sci Rep. 2015 Sep 1;5:13637. doi: 10.1038/srep13637 (PMC5378903; doi:10.1038/srep13637)
Supplement: Supplementary Table S1 X Figure S1 [file srep13637-s1.pdf]

Selective *in vivo* and *in vitro* activities of 3,3′-4-nitrobenzylidene-bis-4-hydroxycoumarin against methicillin-resistant *Staphylococcus aureus* by inhibition of DNA polymerase III

Zheng Hou[1, #], Ying Zhou[1, #], Jing Li[2,#], Xinlei Zhang[3], Xin Shi[1], Xiaoyan Xue[1], Zhi Li[1], Bo Ma[1], Yukun Wang[1], Mingkai Li[1,*] and Xiaoxing Luo[1,*]

[1]Department of Pharmacology, School of Pharmacy, Fourth Military Medical University, Xi’an, Shaanxi, 710032, China.

[2]School of Chemistry and Chemical Engineering, Xi’an University of Arts and Sciences, 710065, Xi’an China

[3]School of Pharmacy, Fourth Military Medical University, Xi’an, Shaanxi, 710032, China.

Footnotes

[#]Z.H., Y.Z. and J.L. contributed equally to this work.

*Corresponding author: mingkai@fmmu.edu.cn (Mingkai Li); xxluo3@fmmu.edu.cn (Xiaoxing Luo); Tel./fax: +86 29 84774591.

All authors declare no conflicts of interest. All experiments were performed in accordance with relevant guidelines and regulations.

Supplementary Table S1 MICs of NBH and antibiotics in Mueller-Hinton Broth Culture.

Drugs	MICs (mg/L)		
	NBH	Oxacillin	Levofloxacin
S. epidermidis ATCC14990	16	<0.25	0.12
MRSE XJ75284	32	>128	8
E. coli ATCC25922	>256	-	<0.25
P. aeruginosa ATCC27853	>256	-	2

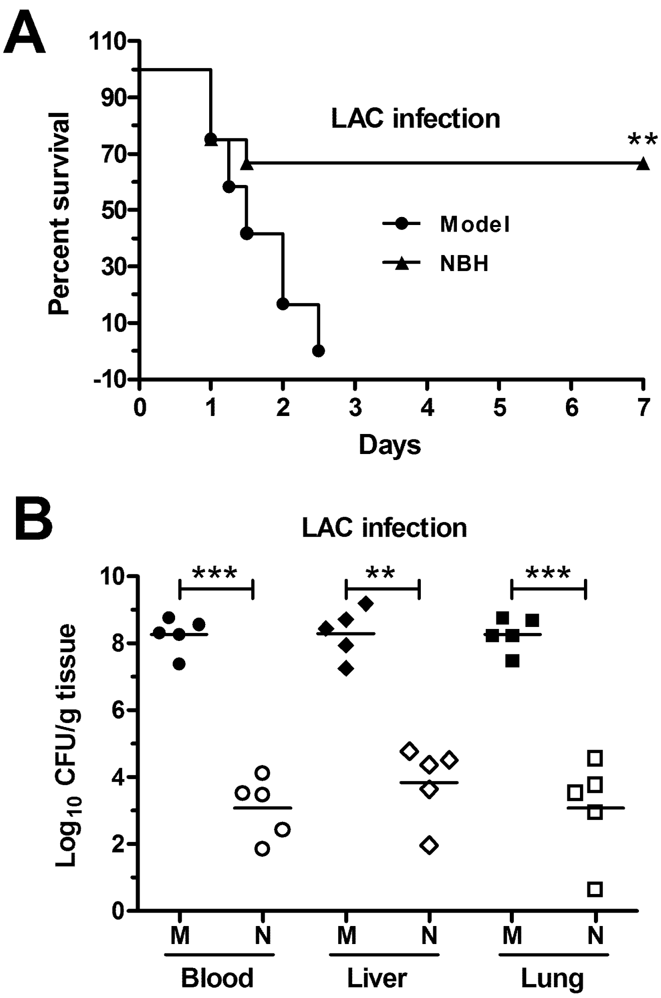

Supplementary Figure S1 Mice (n = 17/group) were infected by the intraperitoneal (i.p.) administration of 5.6×10^8 CFUs LAC inoculum in 0.4 mL of Mueller-Hinton broth. After bacterial challenge for 1 h and 6 h, mice were randomized to receive 5 mg/kg NBH (20 ml/kg, containing 0.9% saline) intraperitoneal injection. To assess bacterial clearance, 5 mice in each group were removed from their cages and 100 μL blood samples for culture were obtained from the tail vein through aseptic percutaneous puncture 24 h after infection. After that, cervical dislocation was performed manually resulting in euthanasia within 10 seconds, and liver and lung were harvested aseptically from these mice, weighed, and homogenized in sterile saline solution. A. Survival of

BALB/c mice (n = 12/group) inoculated by i.p. injection with LAC and treated with NBH at 5 mg/kg containing 0.9% saline by i.p. administration at 1 and 6 h after infection. B. Colonisation of LAC inoculum in the liver, lung and blood cultures of NBH-treated BALB/c mice (n = 5/group) 24 h after infection. **P <0.01, ***P <0.001 versus Model.
